# Supplementary material for: Biofilm Formation by Escherichia coli Isolated from Urinary Tract Infections from Aguascalientes, Mexico
Source: Microorganisms. 2023 Nov 25;11(12):2858. doi: 10.3390/microorganisms11122858 (PMC10745304; doi:10.3390/microorganisms11122858)
Supplement: Supplementary file 1 [file microorganisms-11-02858-s001.zip › microorganisms-2705874-supplementary.pdf]

Supplementary Table S1. Oligonucleotides and PCR conditions used in this study.

Target gene	Sequence	Amplification size (pb)	PCR condition	Ref.
uidA	F: 5′-AATGTGCTGTGCCTGAACC-3′<br>R: 5′-ATTGTTTGCCTCCCTGCTG-3′	450	94ºC 5 minutes; 30 cycles of 94ºC for 30 seg, 54ºC for 30 seg, 72ºC for 30 seg; final extension 72ºC for 7 min.	[43]
agn43	F: 5′- TTCCGGGAAGACGGTGAA -3′<br>R: 5′-TTCTGGGTGAGTGTGGTGTTG-3′	144	94ºC 5 minutes; 30 cycles of 94ºC for 30 seg, 56ºC for 30 seg, 72ºC for 30 seg and; a final extension 72ºC for 7 min.	[49]
afa/dra	F: 5′-GGCAGAGGGCCGGCAACAGGC-3′<br>R: 5′-CCCGTAACGCGCCAGCATCTC-3′	592	94ºC for 5 minutes; 30 cycles of 94ºC for 30 seg, 63ºC for 45 seg, 72ºC for 45 seg and; a final extension 72ºC for 7 min.	[45]
papC	F: 5′-GACGGCTGTACTGCAGGGTGTGGCG-3′<br>R: 5′-ATATCCTTTCTGCAGGGATGCAATA-3′	350	94ºC for 10 minutes; 30 cycles of 94ºC for 30 seg, 56ºC for 1.5 min, 72ºC for 1.5 min and; a final extension 72ºC for 10 min.	[45]
hlyA	F: 5′-AACAAGGATAAGCACTGTTCTGGCT-3′<br>R: 5′-ACCATATAAGCGGTCATTCCCGTCA-3′	1117		[47,48]
cnf1	F: 5′-AAGATGGAGTTTCCTATGCAGGAG-3′<br>R: 5′-CATTCAGAGTCCTGCCCTCATTATT-3′	498		[45]
sfaS	F: 5′-GTGGATACGACGATTACTGTG-3′<br>R: 5′-CCGCCAGCATTCCCTGTATTC-3′	240	94ºC for 10 minutes; 30 cycles of 94ºC for 30 seg, 54ºC for 1 min, 72ºC for 1 min and; a final extension 72ºC for 10 min.	[45]
fimH	F: 5′-TGCAGAACGGATAAGCCGTGG-3′<br>R: 5′-GCAGTCACCTGCCCTCCGGTA-3′	508		
kpsMTII	F: 5′-GCGCATTTGCTGATACTGTTG-3′<br>R: 5′-CATCCAGACGATAAGCATGAGCA-3′	272		
fyuA	F: 5′-TGATTAACCCCGCGACGGGAA-3′<br>R: 5′-CGCAGTAGGCACGATGTTGTA-3′	880	94ºC for 15 minutes; 30 cycles of 94ºC for 30 seg, 63ºC for 1.5 min, 72ºC for 1.5 min and; a final extension 72ºC for 10 min.	[45]
yfcV	F: 5′-ACATGGAGACCACGTTCACC-3′<br>R: 5′-GTAATCTGGAATGTGGTCAGG-3′	292		[46]
vat	F: 5′-TCAGGACACGTTCAGGCATTCAGT-3′<br>R: 5′-GGCCAGAACATTTGCTCCCTTGTT-3′	550		[46]

Single and multiplex PCR were used in order to detect virulence genes. Genes tested are listed following the singleplex or multiplex PCR used in the study.

Supplementary Table S2. Function of virulence markers for UPEC tested in this study.

VF type	Target gene	Name	Function	Ref.
Adhesins	*fimH*	D-mannose specific subunit, FimH, type 1 fimbriae	Participate in UPEC adherence, colonization and invasion of the bladder epithelium which is critical for lower UTIs. Binds mannosylated glycoproteins on superficial epithelial cells in the bladder. Facilitates the biofilm formation. Also mediate binding to the intestinal crypts, helping to establish a stable gastrointestinal reservoir.	[5,31, 43]
	papC	P fimbriae chaperone	Stimulate the production of cytokines by T lymphocytes and is a colonization factor highly expressed in pyelonephritis in order to attach to digalactoside receptor that are expressed on the kidney epithelium. Pili bind globosides in the kidney and are essential for progression to pyelonephritis.	[5,50-52]
	yfcV	Major subunit of a putative chaperone-usher fimbriae	Related to more efficiently colonization of urinary tract.	[46]
	agn43	Phase-variable outer membrane protein adhesin antigen 43	Adhesin of the autrotransporter (AT) family. Mediates cell aggregation, adhesion, biofilm aggregation and IBCs formation and, enhancing colonization and persistence in the urinary bladder.	[6,49]
	afa/dra	Dr-binding adhesins	Afa is a non-fibrous adhesin binds to the decay-accelerating factor (DAF) receptor on the cell surface epithelium and is related to hemagglutination capacity. *dra* encodes the Dr fimbriae that has the function to binding to the DAF receptor on the surface epithelial cells and mediate the internalization bacteria to the host cells.	[52]
	sfaS	Pilus tip adhesin, S fimbriae	Participate in the adhesion to intestinal epithelial cells, kidney and lower urinary tract cells. Facilitate the penetration of bacteria into the tissues.	[52]
Toxins	*hlyA*	α-haemolysin	Damage the renal epithelium by formation of pores. Contribute to activation of the host inflammasome by activating caspase 1 and 4, leading to urothelial cell death.	[5,50]
	cnf1	Cytotoxic necrotizing factor 1	Contribute to the inflammation and tissue damage.	[45]
	vat	Autotransporter serine protease toxin	Vat induced vacuole formation on the urothelium model, cellular damage and change in the cytoskeletal including distribution of F-actin and tubulin.	[52,51]
Siderophores	*fyuA*	Yersiniabactin receptor	Outer membrane-protein receptor for internalization. Associated with UPEC virulence in the bladder. Required for the establishment of septicemia.	[46,50]
Capsular synthesis	*kpsMTII*	Group II capsular polysaccharide synthesis	Protection factor against phagocytosis.	[45-46]

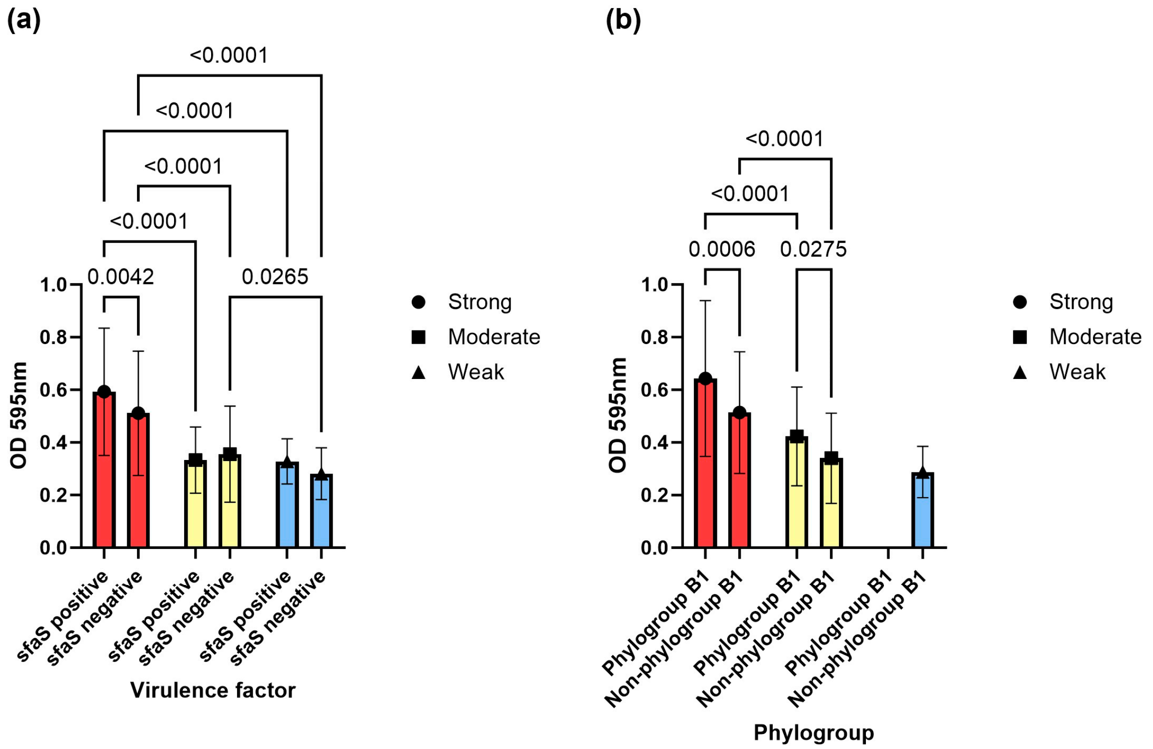

Supplementary Figure S1. Comparison among optical densities (OD) among strong, moderate and weak biofilm formation in strains positive and negative for (**a**) *sfaS* gene and (**b**) phylogroup B1. Comparison among optical densities of strong-, moderate-, and weak-biofilm-producing strains was performed using the non-parametric two-way analysis of variance (ANOVA) with a Tukey´s multiple comparisons post-hoc test.
